# Ovarian carcinoma glyco-antigen targeted by human IgM antibody

**DOI:** 10.1371/journal.pone.0187222

**Published:** 2017-12-21

**Authors:** Yi Chen, Marcia M. Bieber, Neelima M. Bhat, Nelson N. H. Teng

**Affiliations:** Department of Obstetrics and Gynecology, Division of Gynecologic Oncology, Stanford University, Stanford, California, United States of America; Columbia University, UNITED STATES

## Abstract

Epithelial Ovarian Cancer (EOC) cells expression of a novel carbohydrate antigen was defined using a human VH4-34 encoded IgM monoclonal antibody (mAb216). MAb216 binds to a poly N-acetyllactosamine epitope expressed on B cells and kills normal and malignant B cells in vitro and in vivo. EOC patient ascites and EOC cell lines were used to study the anti tumor effect of mAb216. Various assays were used to characterize the epitope and demonstrate antibody-mediated binding and cytotoxicity in EOC. Drug and antibody combination effects were determined by calculating the combination index values using the Chou and Talalay method. MAb216 displays direct antibody mediated cytotoxicity on a population of human EOC tumor and ascites samples and EOC cell lines, which express high amounts of poly N-acetyllactosamine epitope, carried by CD147/CD98. Eighty four percent of patient samples, including platin resistant, had a tumor population that bound the monoclonal antibody. The binding pattern of mAb216 and mechanism of cytotoxicity was similar to that seen on normal and malignant B cells with unique general membrane disruption and “pore” formation. In vitro incubation with mAb216 and cisplatin enhanced killing of OVCAR3 cell line. In EOC cell lines percent cytotoxicity correlated with percent expression of epitope. Although in vitro data shows specific EOC cytotoxicity, for possible treatment of EOC MAb216 would need to be evaluated in a clinical trial with or without chemotherapy.

## Background

MAb216 is a human derived IgM monoclonal antibody (mAb) encoded in germline configuration by the immunoglobulin VH4-34 heavy chain gene. VH4-34 encoded antibodies are found in plasma at low levels in normal subjects and are detectable only in certain clinical conditions such as EBV infection, SLE, HIV [1]. Many mAbs derived from the VH4-34 heavy chain gene bind to a distinct straight chain poly n-acetyl lactosamine, referred to as the i/I antigen [2,3] and found on normal fetal red blood cells (RBCs) and mature B cells [4]. On glycan array (Consortium for Functional Glycomics), these VH4-34 encoded antibodies bind a long straight chain, poly N-acetyllactosamine with or without a terminal sialic acid [5]. Recently by using immuno-precipitation and Mass Spectrometry (Nano ESI), our group further identified the protein carrier of straight chain poly n-acetyl lactosamine epitope on B cell (including normal human B cells and human B cell lines) membrane as CD147/CD98 complex. CD45 on human normal B cells also carry this carbohydrate structure [5,6].

MAb216 binds and kills normal and malignant B cells in vitro and in a xenogeneic model of acute leukemia [7]. MAb216 was evaluated in a National Cancer Institute (RAID program) phase I trial in patients with relapsed or refractory B-cell acute lymphoblastic leukemia. MAb216 was well tolerated and combination with vincristine showed efficacy and no grade 3 toxicity [8].

To further explore the mAb216 applications and expression in solid tumors, we examined mAb216 epitope (straight chain poly n-acetyl lactosamine epitope) expression in epithelial cancer cells using established cell lines from certain diseases. MAb216 and several other similar VH4-34 encoded IgM mAbs bound to some cell lines derived from ovarian carcinoma, breast cancer and glioblastoma, among those examined. However, it is important to establish that mAbs bind to patient tumors, not just cell lines. As we had access to epithelial ovarian carcinoma [EOC] patient samples including cancer cells from both primary and recurrent patients we focused on this tumor. Although aberrant glycosylation has been reported in EOC this is the first report of a defined poly N-acetyllactosamine antigen and recognition by a human IgM mAb

Epithelial Ovarian Cancer (EOC) is the fifth most common cause of cancer in women worldwide bearing the highest mortality rate among all gynecologic cancers. Current first line chemotherapy for EOC is platinum and taxane combination and response rate is high around 70–80%. However, 85% of the patients will have recurrent disease developing resistance to chemotherapeutic agents (9, 10). Other treatments such as targeted therapy anti-VEGF and PARP inhibitor in BRCA mutated patients, have had success, but the overall survival and disease free survival in recurrent EOC patients have not been significantly improved [9,10]. Therefore, more effective therapeutic modalities are urgently needed. In this study, we describe human IgM mAb216 targeting its glycosylation epitope on EOC samples and cell lines, and the unique mode of cytotoxicity

## Methods and materials

### Patient samples and EOC cell lines

Patient ascites removed at surgery or paracentesis were centrifuged and frozen in fetal calf sera and DMSO in liquid nitrogen. Tumor was mechanically dissociated washed and frozen as above. Signed informed consent was obtained from all subjects for specimen collection and the Committee for the Protection of Human Subjects at the Stanford University School of Medicine approved this study, protocol 13939.

NCI ovarian carcinoma cell lines were OVCAR3 (ATCC HTB 161) and OVCAR5 (NCI-DTP). Kuramochi cell line (JCRB cell bank 0098) was a gift from Oliver Dorigo M.D. and was not validated. Human epithelial ovarian cancer cell lines were cultured in Iscove’s media with 10% fetal calf serum (FCS). OVCAR3 and OVCAR5 spheroids were grown in non-adherent flasks in stem cell media with fibroblast growth factor (Essential 8 Basal Medium, with growth factors, Gibco).

### Antibodies

VH4-34 encoded monoclonal antibody MAb216 (IgM) is purified from hybridoma [11] and labeled with-Alex Fluor 488 (Alexa Fluor 488 Protein Labeling Kit, Invitrogen) or n-hydroxysuccinimide biotin. Labeled monoclonal antibodies and streptavidin (SA) were purchased from BD Bioscience or Biolegend. Paclitaxel (#T7402, Sigma) and Cisplatin (#sc-200896, Santa Cruz Biotechnology) were dissolved in DMSO.

### Immunofluorescence

**Fig 1:**
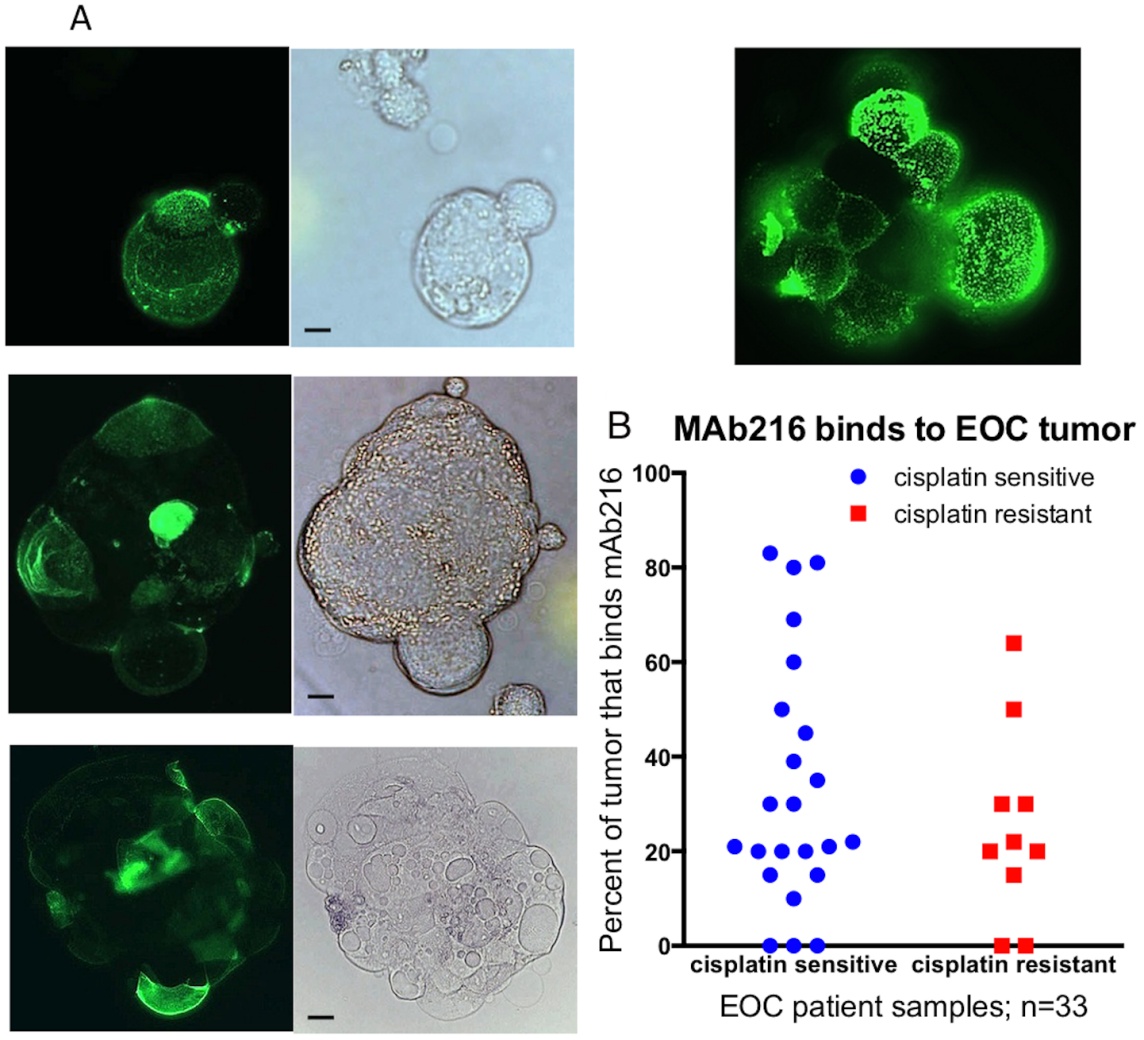
MAb216 binds to high-grade EOC patient’s ascites. (A) Immunofluorescence staining of mAb216-labeled 488 binding to 4 individual high-grade EOC patients’ ascites cells. Two of the ascites clumps are spheroid like. MAb216 binds to the EOC cell surface with a distinct speckled staining pattern. Bar equals 5 microns. (B) Percent of epcam positive or tumor cells staining positive for mAb216 from 33 individual high-grade EOC ascites or tumor samples.

Fresh or frozen EOC tumor or ascites (n = 33, Fig 1) stained with anti CD45-PE and mAb216-488 or anti epcam 488 and mAb216 biotin, streptavidin PE and examined by immunofluorescence using an Axioplan 2 Microscope (Carl Zeiss, Inc., GmbH) equipped with AxioCam HRc camera interfaced with Axiovision 3.1 software, for counting and photography. To determine percent cells binding mAb216 and obtain pictures of mAb216 binding pattern, the percentage of mAb216 positive tumor cells was calculated using CD45 negative and epcam positive, mAb216 positive cells and an estimate of percent mAb216 positive determined. In some samples, epcam was very weak/negative and tumor was determined by morphology. At least 200 cells in three different microscope fields were counted. Percent epcam positive and CD45 positive was also determined. Many tumor samples studied using immunofluorescence were also analyzed by flow cytometry, described below. As has previously been described in EOC there was wide variation in percent tumor in the ascites sample and intensity of epcam stain. The amount of the various cell types per volume of ascites fluid was not noted.

### MAb216 cytotoxicity assay

Frozen ascites or dissociated tumor (n = 7) was washed and incubated overnight at 37° in Iscove media with 5% FCS. Incubation was done in round bottom tubes at a cell concentration of 1x10^6^ per 200μl. MAb concentration was 50μg/ml. Another tumor incubation protocol was for 2 hours at 4 degrees. Cells were stained with anti epcam-488, anti CD45 APC and Propidium iodide (PI) for viability. In a tube separate from incubation, ascites cells were stained for mAb 216 binding using mAb216 biotin-SA-APC and epcam 488. Cells were analyzed by flow cytometry at the Stanford Flow Cytometry Center on a LSR II model 1A analyzer, a 4 laser 14-color instrument, (Becton Dickinson). Not all ascites samples could be used for cytotoxic flow cytometry analysis as some consisted of very large clumps that could not be dissociated.

Using the control sample, cells were counted for a set time applied to all samples or Sphero Accucount Particles (Spherotech, Inc) were added to the samples to determine loss of dead cells. In all analysis, dead cells and debris were removed after data acquisition by gating on control sample forward scatter and PI negative using Flowjo (Treestar, Ashland, OR) software. Subpopulation gates were usually determined using Flowjo ‘cluster’ software (S1 Fig) and number of live cells in the treated samples determined. For mAb216 staining of samples analyzed both by microscopy and flow cytometry, percent of mAb216 positive tumor cells was higher with flow cytometry.

### Ovarian carcinoma cell line studies

Ovarian carcinoma cell lines were stained with mAb216-488 either as adherent cells or after trypsinization as a cell suspension when used for flow cytometry. (The glycosylation epitope is not affected by trypsin treatment). Stained slides were examined by immunofluorescence microscopy. Trypsinized cells were analyzed by flow cytometry. Cell lines were incubated with MAb216 for cytotoxicity as described for ascites samples.

### Flow cytometry sorting of mAb216 high-binding and low-binding OVCAR3 populations

For cell sorting, 5x10^7^ OVCAR3 cells were stained with 50μg of mAb216-Alex Fluor 488 [less than saturation concentration] and PI for 1 hour at room temperature, washed three times with PBS, and sorted on a BD Aria flow cytometer at Stanford FACS facility. FCS files are available from the Stanford FACS facility

### Treatment of OVCAR3 with Endo-beta-Galactosidase

OVCAR3 cells 1x106 were washed and suspended in PBS. 10mU Endo-beta-Galactosidase (*Escherichia freundii*) was added and incubated for 1 hour at 37°. After washing, treated and untreated controls were stained with mAb216-488 for flow cytometry. Endo-beta-Galactosidase (*Escherichia freundii*) was made by Seikagaku Chemicals and purchased from Amsbio LLC. Although having the same EC number this enzyme is not the same as keratanase.

### Immunoprecipitation and Western blot

OVCAR3 cells were processed as described in [5] and in the S1 Protocol and the detergent insoluble extract electrophoresed and blotted. Detection was with anti-CD147 (F-5, sc 374101), and anti-CD98 (E-5, sc 21746), (Santa Cruz Biotechnology).

### Scanning electronic microscopy

OVCAR3 cells in staining medium were incubated with mAb216 (100 μg/ml, maximum saturation of mAb to induce extensive membrane perturbation) or control staining medium for 1 hour at room temperature or 4°C, washed with PBS and then fixed with 2% glutaraldehyde in 0.1M sodium phosphate buffer pH 7.3 at 4°C overnight. The fixed cells were washed with buffer, post-fixed for 1 hour in 1% OsO4, washed with distilled water, dehydrated in 30–100% ethyl alcohol and dried in hexamethyldisi-lazane. Cells were mounted onto a double sticky Pelco carbon conductive stub. The stub was gold-coated on a Polaron 5300 for 5 min, and examined on a Philips 505 Scanning Electron Microscope (Philips, Einhoven, Holland) located at Stanford Cell Science Imaging Facility.

### Cell cytotoxicity assay for drug interaction

Cells were plated in 96-well micro plates at a density of 8x10^3^ cells per well for 24 hours before drug application. Experimental drugs are solubilized in dimethyl sulfoxide. 72 hours after drug application, cells were fixed by ¼ volumes of cold 50% TCA incubation, followed by staining with 50μl of 0.4% Sulfarodamine B solution in 1% acetic acid for 30 minutes. Processing followed manufacturer’s instructions (Sigma). The plate was read at 515nm.

Dose-response curves are constructed and drug combinations effects evaluated by the Combination Index-Isobologram Theorem (Chou-Talalay) using the CompuSyn software (ComboSyn, Inc., Paramus, NJ). Drug interactions were quantified by determining the combination index (CI), where CI<1, CI = 1, and CI>1 indicate synergistic, additive, and antagonistic effects, respectively.

### Statistics

Statistical analysis was performed with Excel and GraphPad Prism5 as needed.

## Results

In this study we used mAb216 a well-described germline IgM VH4-34 encoded mAb, which as been studied in vivo. On B cells immunostaining with IgM VH4-34 mAbs gives a characteristic speckled or dotted pattern (14, 15). In thirty-three samples of mostly high grade EOC mAb216 bound to a subset of tumor cells with this distinct dotted staining pattern (Fig 1A). Epcam was used as the marker for tumor in most samples analyzed. Binding of mAb216 ranged from 10–80% of epcam positive or tumor cells as determined by fluorescence microscopy and/or flow cytometry (Fig 1B). Only five patients (15%) showed no mAb216 binding. There are no statistical differences in mAb216 binding between patients with cisplatin sensitivity/resistant.

Incubation of seven tumor samples with mAb216 at 37°, lead to cell death of a percent of epcam positive tumor cells (Table 1 and S1 Fig). The percent killed correlated with the percent of tumor cells that bound mAb216. As previously seen with B cell incubation, (14, 15) incubation at 4° also leads to enhanced vitro cytotoxicity of the patient’s EOC cells that bind mAb216.

**Table 1:** Incubation Of EOC ascites tumor cells with mab216 causes cell death.

OVCA Ascites sample	Percent of ascites cells that are epcam positive	Percent of epcam positive cells that are mAb216 positive	*Percent decrease in live epcam positive cells post Rx with mAb216 at 37°	*Percent decrease in live epcam positive cells post Rx with mAb216 at 4°
G	54%	69%	43%	65%**
M	16%	83%	36%	80%**
K	44%	21%	14%	24%**
R	51%	71%	20%	76%**
W	4%	30%	15%	18%
B	79%	39%	10%	29%
C	25%	35%	22%	ND
F	7%	20%	0%	22%**

Frozen ascites cells were washed and incubated overnight at 37°with mAb216 50μg/ml or no addition or for 2 hours at 4 degrees. Cells were stained for epcam, CD45 and PI and analyzed by flow cytometry using timed collection or beads to detect cell loss.

*Percent decrease equals: Control [number of epcam positive, PI negative cells] minus [mAb216 treated number of epcam positive, PI negative cells] divided by control cells.

**All tumor cells that bound mAb216 [column 2 staining] either lysed or were dead [PI+] after mAb treatment.

MAb216bio-SA-APC positive cells were determined in a separate tube. [See Material and methods and S1 Fig].

We evaluated Kuramochi cell line and six NCI-60 EOC cell lines: OVCAR3, OVCAR4, OVCAR5, OVCAR8, SKOV3, IGROV, for mAb216 binding. These lines were obtained from the NCI Developmental Therapeutics Program, https://dtp.cancer.gov. The DTP Cellminer site; https://discover.nci.nih.gov/cellminer has complete information on origin and the molecular and pharmacological data sets for the NCI-60 cell lines. OVCAR3 cells showed 15–25% mAb216 positive cells, while OVCAR5 and Kuramochi had 4–10% positive. The other lines did not bind mAb216 in adherent culture. Based on gene expression, IGROV and SKOV3 may not represent serous EOC [12]. The distinct speckled staining pattern was seen on mAb216 positive EOC cell lines using immunofluorescence. As OVCAR3 showed the same mAb binding pattern as seen on EOC patients’ samples it was used for in vitro studies.

**Fig 2:**
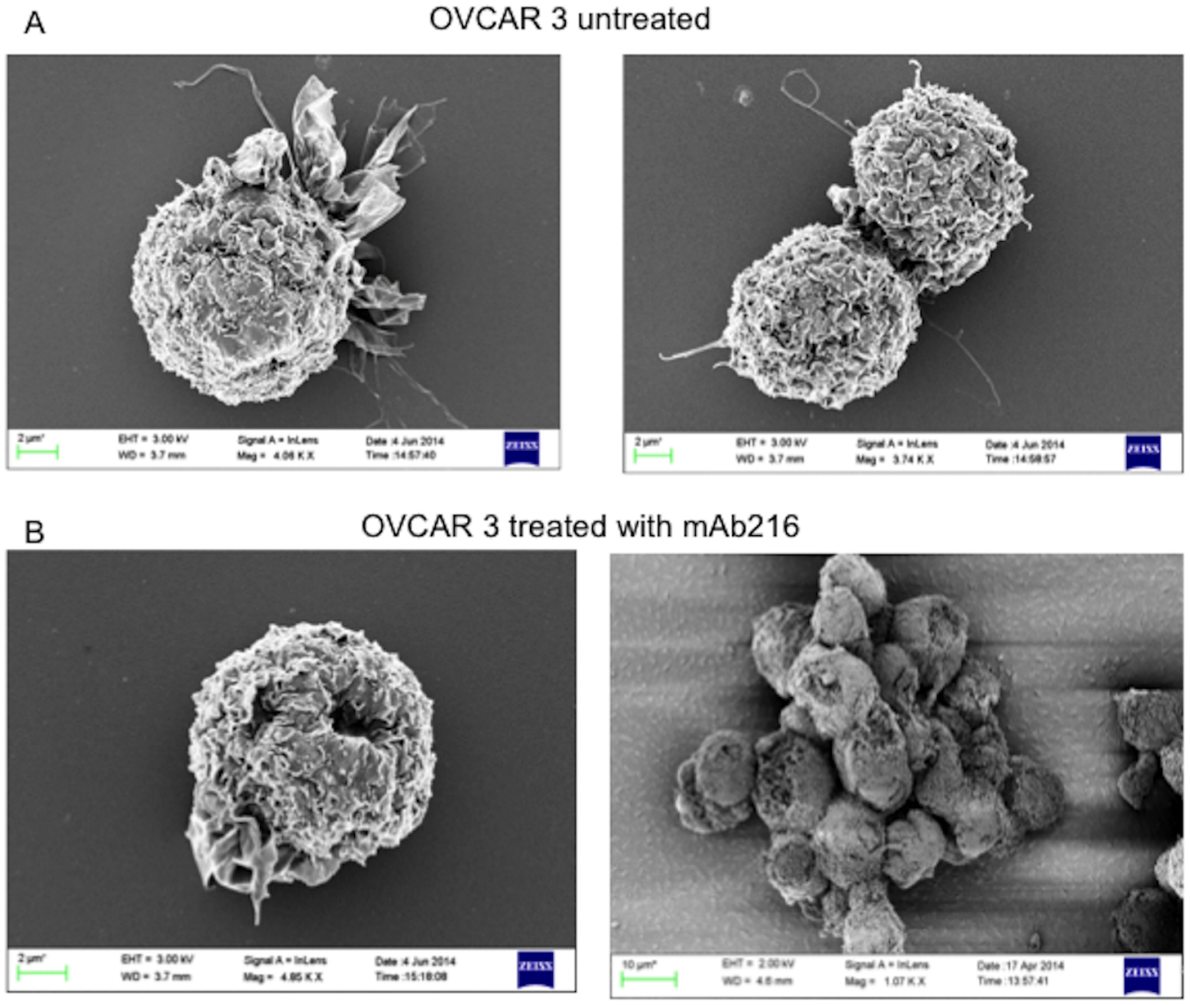
MAb216 binding leads to unique membrane disruption. OVCAR3 cells were incubated with mAb216 (100 μg/ml) [B] or control staining medium [A] for 1 hour at room temperature. Fixed cells were examined by scanning electron microscopy. Treated cells [B] show membrane damage and "pore" formation.

Scanning electron microscopy (SEM) shows pore like structures on treated B cells [13]. SEM of OVCAR3 shows cell agglutination, general membrane disruption and pore like structures after mAb216 treatment, similar to previous described mAb216 treated B cells (Fig 2).

**Fig 3:**
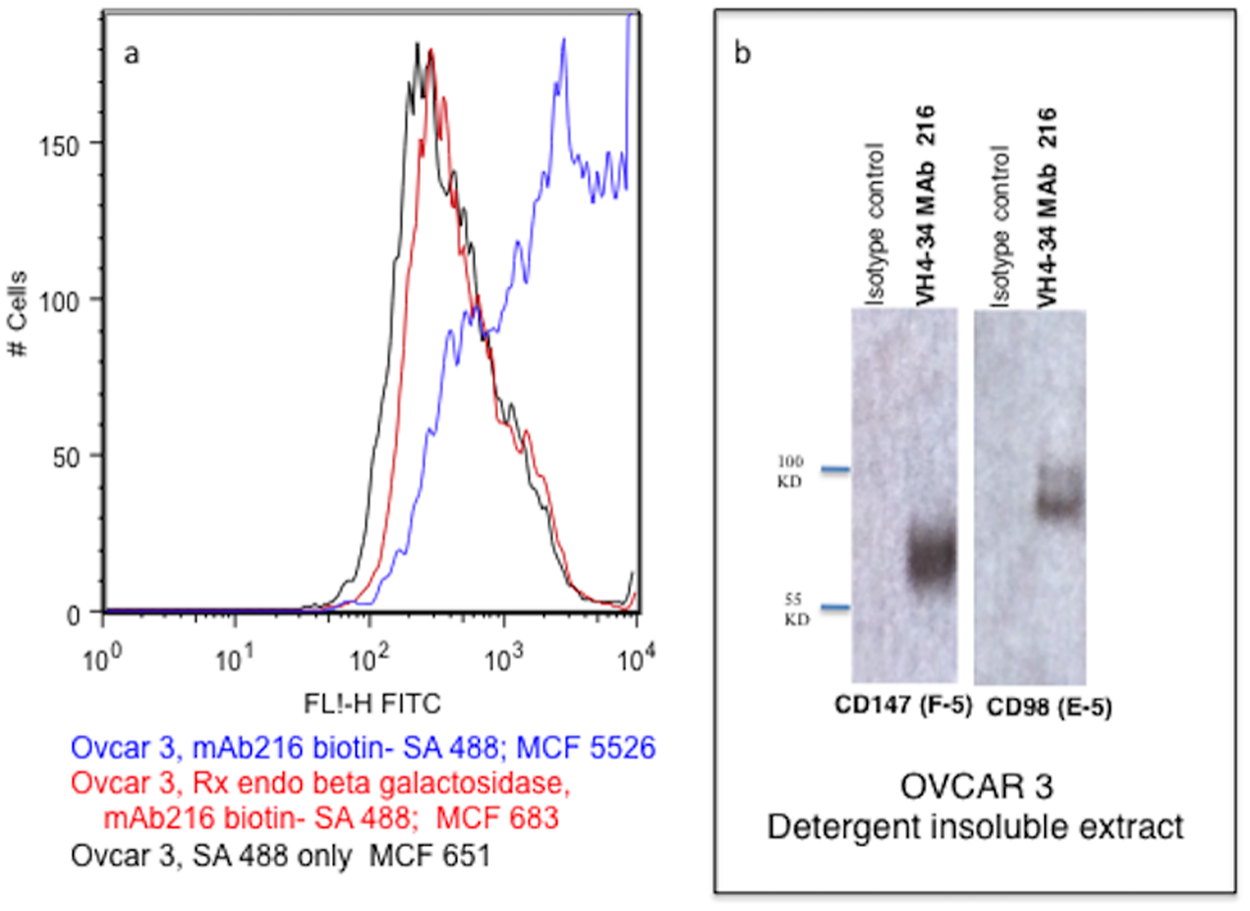
Characterization of the epitope recognized by mAb216 on ovarian carcinoma. [a] OVCAR3 spheroids treated with endo beta galactosidase and untreated stained with mAb216 biotin SA-488. The enzyme removes poly N-acetyllactosamine antigen. Also shown are OVCAR3 spheroids stained with SA-488 only as control for mAb216 biotin SA-488 staining. [b] MAb216 precipitates a highly glycosylated CD147 and CD98 complex from OVCAR3.

Further evidence that the poly N-acetyllactosamine epitope is the same on EOC and B cells was shown by removal of the epitope [and mAb216 binding] by endo beta galactosidase on OVCAR3 cells (Fig 3a). Endo beta galactosidase cuts the bonds between interior galactose and n-acetylglucosamine and the hexose chain is shortened indicating that the intact poly N-acetyllactosamine structure is critical for the binding and killing.

Immunoprecipitation with mAb216 of the detergent insoluble extract of OVCAR3 and detection on Western with anti CD147 and anti CD98 mAbs showed the epitope was carried by the highly glycosylated CD147-CD98 complex, as seen on malignant B cells [5]. (Fig 3b)

MAb216 binding to poly N-acetyllactosamine expressing EOC cell lines OVCAR3 and OVCAR5 leads to cytotoxicity in vitro (Table 2). MAb216 binding to OVCAR3 and OVCAR5 differs between attached cells and spheroids form [cultured in stem cell media]. OVCAR3 cultured as spheroids [14] shows a higher percent of mAb216 positive cells (50–60%) than attached culture (15–25%) with an increase in percent of cell death when incubated with mAb216. Similar results were obtained with OVCAR5 (Table 2). In vitro cytotoxicity with cell lines further confirms that mAb216-mediated killing is not dependent on complement and ADCC.

**Table 2:** Incubation Of EOC cell lines with mAb216 causes cell death Of mAb216 binding cells.

Cell Line	Percent cells mAb216 positive	Percent live cells post Rx with mAb216
OVCAR5 (37°)	6	no change
OVCAR5 spheroids (37°)	36	71
OVCAR5 (4°)	8	88
OVCAR5 spheroids (4°)	30	69
OVCAR3 (37°)	15	82
OVCAR3 spheroids (37°)	55	61
OVCAR3 (4°)	21	85
OVCAR3 spheroids (4°)	52	56

Trypsinized adherent or spheroid cultured cells were incubated at 4° for 2 h. or overnight at 37° and analyzed on flow cytometry. Treated live cells shown as percent of untreated control. A separate tube was stained with mAb216 labeled with 488 to determine percent positive.

**Fig 4:**
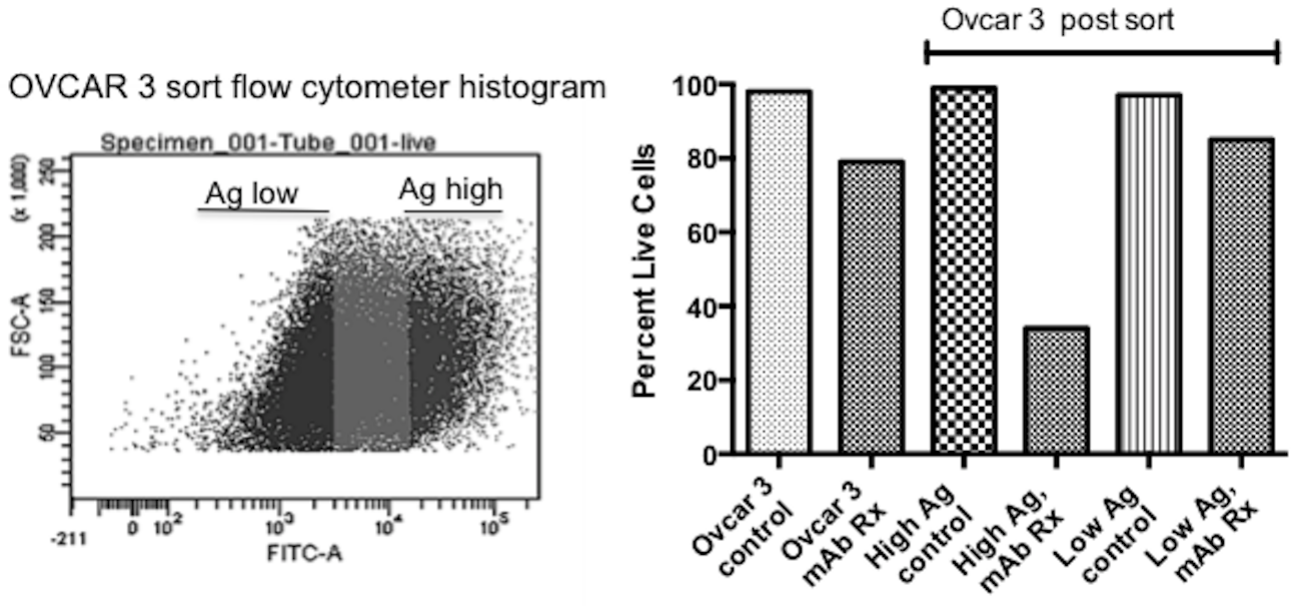
MAb216 cytotoxicity relates to antigen expression. **(A)** Histogram of flow cytometry sorting of OVCAR3 into mAb216 high-binding cells (antigen high expression) and low-binding/negative cells (antigen low expression). (B) The percentage of live cells after treatment with MAb216 (100μg/ml, 3 hours at 37°C) on unsorted OVCAR3 cells (left two columns), sorted antigen high expressing cells (middle two columns) and antigen low cells (right two columns). See Material and methods.

To further show that epitope expression correlated closely with mAb216 killing, we sorted mAb216 high-binding and low binding sub-population by staining with mAb216 at very low concentration to maintain cell viability (Fig 4a). Following the sort we treated both populations with 100μg/ml mAb216 leading to cytotoxicity in 70% of mAb216 binding cells and no toxicity to low binding sub-population, indicating that the level of epitope expression on the cell surface correlates with degree of cytotoxicity (Fig 4b). Interestingly, mAb216 staining of the positive sorted population and negative population over time showed that the negative population re-expressed the epitope by 10 days indicating that epitope re-expression occurs as cells grow. Tumor cells continually re-expressing high amounts of epitope will lead to additive cytotoxicity by each subsequent dosing of antibody. The sorted mAb216 high-binding population and low-binding population showed no difference in cell proliferation rate and drug sensitivity to both paclitaxel and cisplatin (S2 Fig)

**Fig 5:**
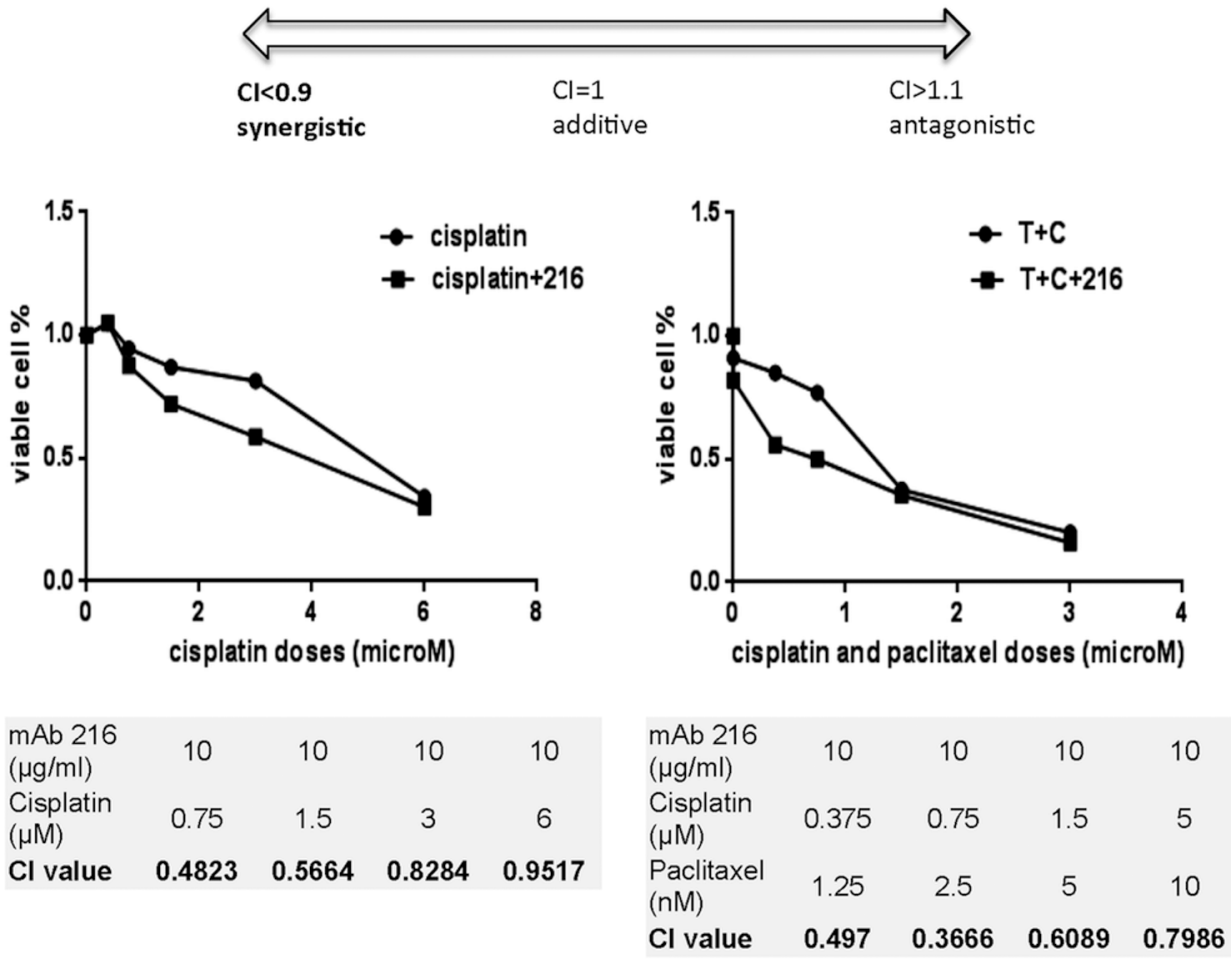
MAb216 synergizes with cisplatin and paclitaxel. OVCAR3 was incubated with cisplatin alone, cisplatin-paclitaxel alone or with mAb216 to determine synergy. Drug combination treatments were designed according to the Chou–Talalay equation, which accounts for both the potency (median inhibitory concentration) and the shape of the dose–effect curve, and the combination index (CI) was determined using CompuSyn software (ComboSyn, Inc.). CI < 1 indicates synergism.

MAb216 can crosslink the carbohydrate ligand, leading to membrane disruption and loss of cell integrity. Cells with high epitope expression will be damaged and break down. However, cells with low epitope expression will initiate the repair mechanism. The addition of secondary drugs (such as chemotherapy drugs) at this time point may synergize with mAb216 in cytotoxicity. Addition of low dose mAb216 (10μg/ml) to cisplatin or a combination of cisplatin and paclitaxel significantly induced OVCAR3 cytotoxicity (Fig 4a and 4b). To determine the drug combination effects, we calculated combination index value (CI value) by software Compusyn, which is based on the Median-Effect Principle and the Combination Index-Isobologram Theorem (Chou-Talalay) (CI <0.9 synergy, CI 0.9–1.10 additive and CI >1.10 antagonism) [15]. Fig 5 shows the CI value of the combination of mAb216 (low dose 10ug/ml) with various concentrations of cisplatin or cisplatin and paclitaxel. All the CI values are less than 0.9. In the lower chemotherapy dose range, mAb216 showed strong synergistic effects with CI value<0.5.

## Discussion

We and others have described binding of human VH4-34 germline encoded antibody to a glycosylation epitope, straight chain poly N-acetyllactosamine, found on B cells. Here we show that human IgM VH4-34 mAb216 binds and kills high-grade EOC samples and ovarian carcinoma cell lines in vitro. Using various methods, we show that the epitope recognized on EOC is similar if not identical to that found on B cells, where it is carried on CD147 and CD45, and on fetal red blood cells (called i antigen). As with normal and malignant B cells mAb216 can kill EOC ascites that express sufficient amount of epitope without complement or ADCC, although IgM can activate the complement pathway. Similar to the B cell cytotoxicity process, arrest of actin in EOC cells by cold incubation leads to rapid cell death, due to inability to repair membrane damage. As seen on B cells, scanning electron microscopy shows that mAb216 crosslinking causes a unique pore like structure on OVCAR3 cells. Removal of the poly N-acetyllactosamine epitope on OVCAR3 cells by endo beta galactosidase abolishes mAb216 binding. Immunohistochemistry analysis shows the epitope is not expressed on normal ovary or normal tissue (S1 File). Aberrant and unique glycosylation can emerge during carcinogenesis and long chain poly n-acetyl lactosamine on tumor cells is said to promote their invasive behavior that ultimately leads to the progression of cancer [16] review in [17]. There is increased interest in glycosylation-based cancer biomarkers or drug targets [18].

Interestingly, the mAb216 epitope expression significantly increased in ovarian cancer cell line spheroid cells compared to the attached cells. Over eight hundred publications indicate spheroid cells share cancer stem cell like characters, such as self-renewal capacity, differentiation potential, tumorigenicity, and increased drug resistance (see PubMed). Although described in ovarian cancer, there are still no definite markers to distinguishing cancer stem cells (22, 23) and reviewed [19]. Increased presence of glyco onco-developmental antigens may be one of the features of cancer stem cell generation. The i antigen (straight chain poly N-acetyllactosamine) found on fetal red cells and on highly glycosylated CD147 on other cells is considered by some to be a stem cell antigen (25, 26). Further studies will clarify whether the mAb216 targeted subgroup of EOC cells carry cancer stem like characteristics.

MAb216 epitope expression on ovarian cancer cells is dynamic. The OVCAR3 flow cytometry sorted low and high antigen cells showed no difference in proliferation. However, as the low antigen subpopulation grew, they re-expressed cells with new epitope expression, similar to the poly N-acetyllactosamine positive percentage in unsorted cells.

Although mAb216 could be used as mono therapy in EOC, the in vitro assay shows it is synergistic with chemotherapy used for treatment of EOC. Cisplatin is the first line chemotherapy for EOC. It binds to and causes crosslinking of DNA, which ultimately triggers apoptosis [20]. Addition of mAb216 to cisplatin or the combination of cisplatin and paclitaxel enhanced cytotoxicity. It remains unclear the exact mechanism but enhanced cytotoxicity is possibly due to mAb216 induced membrane perturbation increasing cisplatin access.

MAb216 is a candidate for treatment of EOC. Based on preclinical animal studies and safety data, the FDA issued an IND for treatment of acute B cell leukemia. MAb216 has been given to 13 patients with B cell acute leukemia without grade 3 toxicity and showed some efficacy, depending on tumor burden, in relapsed and heavily treated patients [8]. Data presented here shows the EOC antigen is equivalent to the poly N-acetyllactosamine epitope on malignant B cells. MAb216 binds and kills normal B cells but not hematopoietic stem cells [21]. Therapy with anti CD20 mAbs has shown that B cell depletion can be managed and the B cells will regenerate. The role of B cells and/or Bregs in progression of EOC and other epithelial cancer is controversial. Future research in the field may define B cell subsets or time points in which B cell depletion is beneficial in epithelial cancers.

In a standard intravenous administration of mAb216 for treatment of EOC, the patient’s B cell population would act as a "sink" requiring a large amount of mAb. However, in EOC chemotherapy (including cisplatin) can be administered intra peritoneal [22] and mAb216 could be delivered through the abdominal port in combination with chemotherapy. The normal peritoneal cavity in human does not contain B cells and although B cells are found in ascites, the amounts are small.

In treating EOC patients, although only a subset of tumor cells (10–80%) could be killed with mAb216 infusion, the tumor cells that re-express high amounts of epitope could be eliminated by subsequent dosing of antibody. MAb216 may also eradicate the subset of drug resistant “stem like” tumor cells if these cells in EOC are defined by the i antigen (straight chain poly N-acetyllactosamine).

MAb216 should be tested in a clinical trial in relapsed EOC

## Supporting information

S1 Fig Flow cytometry gating example for cytotoxicity assay. (PPTX)

S2 Fig Expression of poly n acetyllactosamine epitope does not effect the cell proliferation rate or drug sensitivity. (PPTX)

S1 Table OVCAR 3 expression of poly n acetyllactosamine changes over time. (DOCX)

S1 Protocol Detergent extraction and Western blot protocol. (DOCX)

S1 File Immunopathology report. (PDF)

S1 Dataset Compusyn report. (PDF)

S2 Dataset FACS data archive 1. (ZIP)

S3 Dataset FACS data archive 2. (ZIP)

S4 Dataset FACS data archive 3. (ZIP)

S5 Dataset FACS data archive 4. (ZIP)

S6 Dataset FACS data archive 5. (ZIP)
