# Supplementary material for: Ovarian carcinoma glyco-antigen targeted by human IgM antibody
Source: PLoS One. 2017 Dec 21;12(12):e0187222. doi: 10.1371/journal.pone.0187222 (PMC5739388; doi:10.1371/journal.pone.0187222)
Supplement: S1 Fig — (PPTX) [file pone.0187222.s001.pptx]

## Slide 1
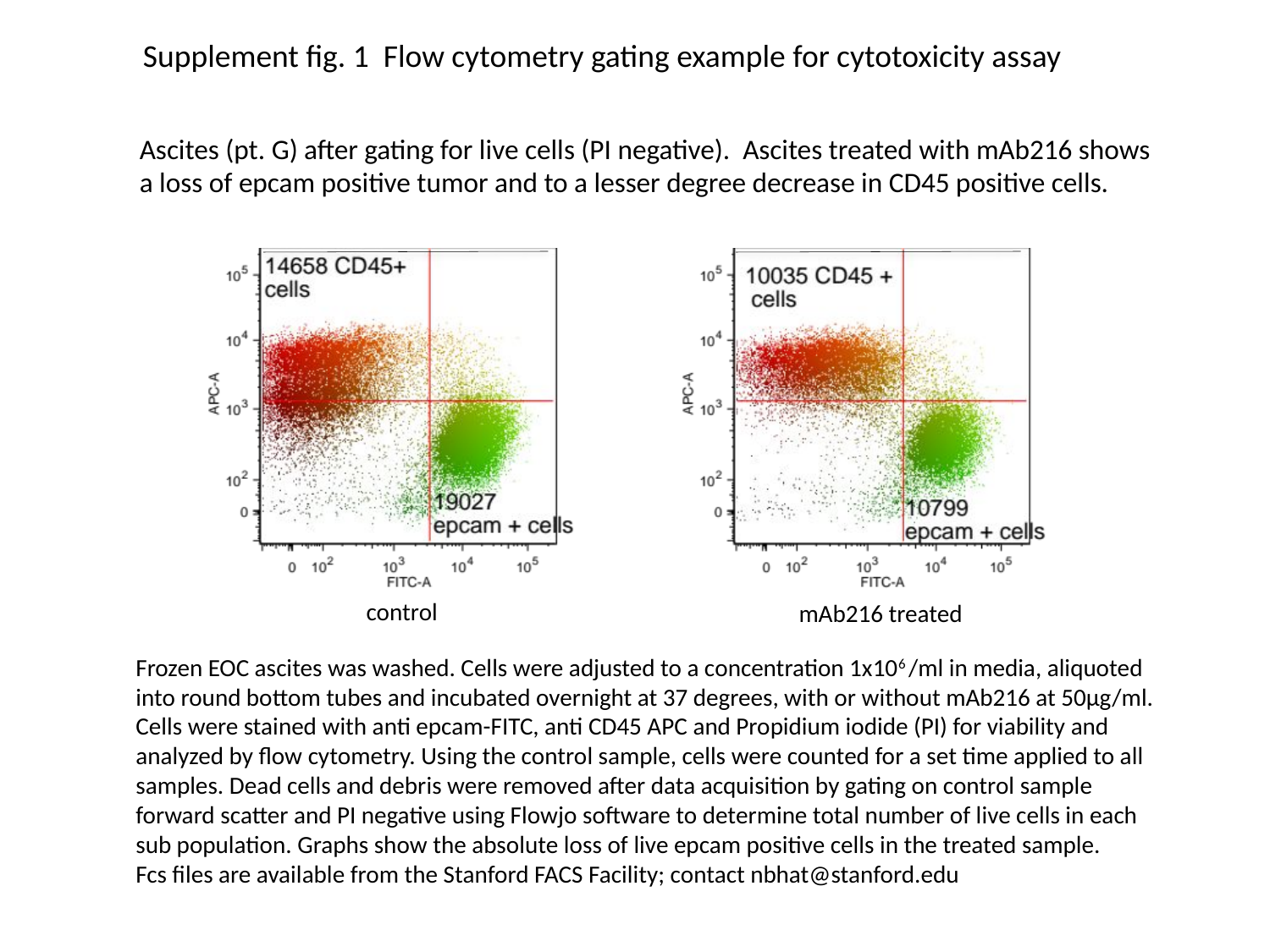

Supplement fig. 1 Flow cytometry gating example for cytotoxicity assay
Ascites (pt. G) after gating for live cells (PI negative). Ascites treated with mAb216 shows a loss of epcam positive tumor and to a lesser degree decrease in CD45 positive cells.
control
mAb216 treated
Frozen EOC ascites was washed. Cells were adjusted to a concentration 1x10^6^ /ml in media, aliquoted into round bottom tubes and incubated overnight at 37 degrees, with or without mAb216 at 50µg/ml. Cells were stained with anti epcam-FITC, anti CD45 APC and Propidium iodide (PI) for viability and analyzed by flow cytometry. Using the control sample, cells were counted for a set time applied to all samples. Dead cells and debris were removed after data acquisition by gating on control sample forward scatter and PI negative using Flowjo software to determine total number of live cells in each sub population. Graphs show the absolute loss of live epcam positive cells in the treated sample.
Fcs files are available from the Stanford FACS Facility; contact nbhat@stanford.edu
