# Supplementary material for: Ovarian carcinoma glyco-antigen targeted by human IgM antibody
Source: PLoS One. 2017 Dec 21;12(12):e0187222. doi: 10.1371/journal.pone.0187222 (PMC5739388; doi:10.1371/journal.pone.0187222)
Supplement: S2 Fig — (PPTX) [file pone.0187222.s002.pptx]

## Slide 1
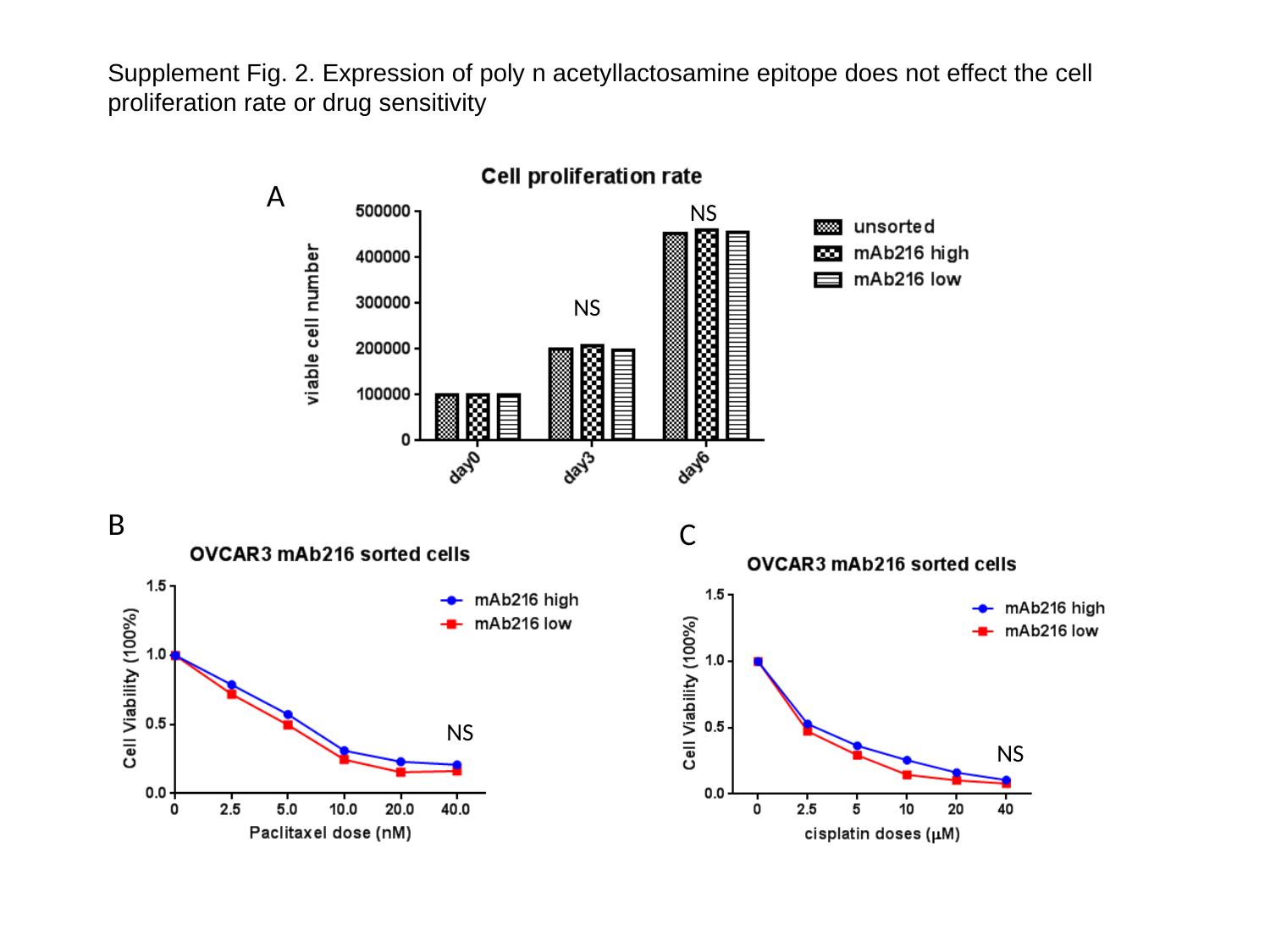

Supplement Fig. 2. Expression of poly n acetyllactosamine epitope does not effect the cell proliferation rate or drug sensitivity
A
NS
NS
B
C
NS
NS
