# Supplementary material for: Ovarian carcinoma glyco-antigen targeted by human IgM antibody
Source: PLoS One. 2017 Dec 21;12(12):e0187222. doi: 10.1371/journal.pone.0187222 (PMC5739388; doi:10.1371/journal.pone.0187222)
Supplement: S1 Table — (DOCX) [file pone.0187222.s003.docx]

Supplement Table 1. OVCAR 3 expression of poly n acetyllactosamine

changes over time

| Days post sort | Day 3 MCF | Day 10 MCF |
| --- | --- | --- |
| OVCAR 3 antigen high; mAb216-488 | 14818 | 16173 |
| OVCAR 3 antigen high; no stain | 1040 | 1230 |
| OVCAR 3 antigen low; mAb216-488 | 4685 | 9252 |
| OVCAR 3 antigen low; no stain | 652 | 908 |
| OVCAR 3 from culture; mAb216-488 | 10185 | 7996 |
| OVCAR 3 from culture; no stain | 701 | 517 |

OVCAR 3 was sorted for high or low antigen expression, see [Figure 3].

Mean channel fluorescence [MCF] detected with mAb216 changes over

time in culture post sort. 'No stain' is background auto fluorescence
