# Supplementary material for: Ovarian carcinoma glyco-antigen targeted by human IgM antibody
Source: PLoS One. 2017 Dec 21;12(12):e0187222. doi: 10.1371/journal.pone.0187222 (PMC5739388; doi:10.1371/journal.pone.0187222)
Supplement: S1 Protocol — (DOCX) [file pone.0187222.s004.docx]

Supplement 1 Protocol

Methods from “Identification of Cell Surface Straight Chain Poly-N-Acetyl-Lactosamine Bearing Protein Ligands for VH4-34–Encoded Natural IgM Antibodies” J Immunol. 2015;195(11):5178-88. (Reference 5)

Immunoprecipitation for detergent insoluble antigen

OVCAR 3 cells were washed with PBS and incubated on ice for 20 min with 30µgs, mAb216, Controls with no antibody or isotype control "Pierce IgM" (myeloma human IgM Thermo Fisher) were set up in parallel. Cells were washed with cold PBS 3X times before extraction with 0.5% NP40 supplemented with protease inhibitors (Roche Diagnostics #04-693-124-001). Extracts were centrifuged at high speed (microfuge 15000 rpm) and the detergent soluble extract stored on ice until use. The detergent insoluble pellet was washed 1X with PBS and incubated for 45min at RT in PBS with DNase-I, DNase-II and RNase A (25µg/ml, Sigma Aldrich, DN-25, D-8764 and R-5000 respectively). Solubilization of nuclear cytoskeletal associated proteins by DNase and RNase treatment has been previously described {Bonvini, 2000 #6; Patton, 1989 #42; Warren, 1988 #43; Huang, 1999 #44}. The solubilized proteins were recovered by centrifugation (microfuge 15000 rpm, 10 min) and termed detergent-insoluble fraction. Both detergent-soluble and insoluble extracts from VH4-34 antibody treated and control specimens were diluted 1:5 in PBS with protease inhibitors and loaded on pre washed CaptureSelect™ IgM Affinity Matrix (Life Technology, BAC BV, #289005). Loaded resin beads were rocked for 2hr at RT, washed 5X with PBS and eluted with 3% SDS in PBS with 2.5mM EDTA. SDS from eluted proteins was removed by detergent removal column (Thermo Scientific, Pierce Biotechnology, #87777).

Gel Electrophoresis and Western Blot

Extracts were separated by SDS-PAGE on 4-12% gradient gels (Novex NP0322BOX, Life Technologies) under reducing conditions and transferred onto PVDF membranes using standard procedures. Membranes were blocked with 5% milk in PBS for 1hr before immune-blotting with primary antibodies followed by secondary anti-mouse IgG HRP diluted 1:2000 in PBS (Cell signaling #7076S). Blots were developed using ECL reagents (Thermo Scientific #1859674 and #1859675) The primary antibodies were anti-CD147 (F-5, Santa Cruz sc-374101 and HIM6, Biolegend #306206), anti-CD45 (H130, Biolegend #304002 and 35-Z6, Santa-Cruz sc-1178),

anti-CD98 (E-5, Santa Cruz sc-376815) and CD298 (Santa Cruz, sc-135998) and ASCT2 (V501, Cell signaling #5345S). The ASCT2, a rabbit polyclonal reagent was detected using anti-rabbit IgG HRP (Promega, W401B).
