# Supplementary material for: Ovarian carcinoma glyco-antigen targeted by human IgM antibody
Source: PLoS One. 2017 Dec 21;12(12):e0187222. doi: 10.1371/journal.pone.0187222 (PMC5739388; doi:10.1371/journal.pone.0187222)
Supplement: S1 File — (PDF) [file pone.0187222.s005.pdf]

Supplement 1

IMMUNOPATHOLOGY REPORT

Cross-Reactivity Study of IgM 216 with Normal Human Tissues

PAI Study No. IM894

Sponsor

National Cancer Institute
Toxicology & Pharmacology Branch, DTP
National Cancer Institute
Executive Plaza North Rm 8038
Bethesda MD 20892-7451

Testing Facility:

Pathology Associates
Division of Charles River Laboratories, Inc.
15 Worman's Mill Court
Suite I
Frederick, Maryland 21701

Jennifer L. Rojko, DVM, PhD
Diplomate, ACVP
Study Pathologist/Study Director

Lisa T. Anderson, DVM
Reviewing Pathologist

SUMMARY

The test article human IgM 216 is a human IgM monoclonal antibody directed against B-cell lymphoma (Bhat et al., 2001). The overall objective was to characterize the potential cross-reactivity of the test article human IgM 216 with normal human tissues. Using an indirect immunoperoxidase method, the test article IgM 216 specifically stained the membrane and cytoplasm of positive control tissue elements (cords of Billroth, marginal zone) in cryosections of human spleen. Reactivity with positive control cryosections was strong to intense at both concentrations of test article examined (10 µg/mL, 2 µg/mL).

IgM 216 did not specifically react with negative control tissue elements in cryosections of human spleen: lymphocytes in the periarteriolar lymphoid sheath (PALS). The negative control antibody human IgM, did not specifically react with positive or negative control tissue elements in cryosections of human spleen. There was no specific reactivity with any tissue elements when the primary antibody (test article or negative control antibody) was omitted from the staining reaction (assay control).

Supplement 1

The test article IgM 216 reacted with mononuclear cells in B-cell regions (follicular mantle lymphocytes in lymphoid tissues, marginal zone in spleen) in humans (expected finding) as well as with mononuclear cells (principally macrophages) and spindloid/dendritic cells in multiple tissues. Some of the macrophage or spindloid/dendritic cell staining may have been mediated by FcμR.

Staining was also observed with nuclei in the majority of tissues, consistent with reports that IgM 216 binds DNA (Bhat et al., 1993). Most of the other IgM 216 cross-reactivities were suggestive of binding to cytoplasm, cytoplasmic granules or cytoplasmic filaments in epithelial, mesenchymal, gonadal, or neural tissues. The cytoplasmic and nuclear reactivities may have limited biological significance. The intact cell membranes in viable cells should restrict access of in vivo administered IgM 216 to the nuclear and cytoplasmic compartments. However, the potential for immune complex disease (binding to cell or nuclear fragments) cannot be eliminated based on these cross-reactivity findings.

SIGNATURES

Jennifer L. Rojko, D.V.M., Ph.D.
Diplomate, A.C.V.P.
Study Pathologist/Study Director
January 23, 2004

Lisa T. Anderson, D.V.M.
Reviewing Pathologist
January 23, 2004
