# Supplementary material for: Ovarian carcinoma glyco-antigen targeted by human IgM antibody
Source: PLoS One. 2017 Dec 21;12(12):e0187222. doi: 10.1371/journal.pone.0187222 (PMC5739388; doi:10.1371/journal.pone.0187222)
Supplement: S1 Dataset — (PDF) [file pone.0187222.s006.pdf]

S1 Dataset

CompuSyn Report

**Experiment Name:**	low 216 triple
**Date:**	101113
**File Name:**	C:\Users\ychen7\Dropbox\Yi\Yi\data\sulfarodamine assay\216\216 with T and C\Low 216 triple drugs 101113.cse
**Description**	

**Drug:**	216 (216) [ug/ml]
**Drug:**	taxol (T) [uM]
**Drug:**	cisplatin (C) [uM]
**Drug:**	T+C (X) [uM]
**Drug Combo:**	216+T (216+T) (216+T)
**Drug Combo:**	216+C (216+C) (216+C)
**Drug Combo:**	216+X (216+X) (216+X)

Data for Drug: 216 [ug/ml]

Dose	Effect
1.56	0.001
3.125	0.002
6.25	0.004
12.5	0.008
25.0	0.016
50.0	0.032
100.0	0.16

7 data points entered.

**X-int:**	2.82504
**Y-int:**	-3.3106 +/- 0.11838
**m:**	1.17189 +/- 0.09461
**Dm:**	668.408
**r:**	0.98409

Data for Drug: T [uM]

Dose	Effect
0.00125	0.174
0.0025	0.287
0.005	0.485
0.01	0.773
0.02	0.807

Dose	Effect
0.04	0.83
0.08	0.855

7 data points entered.

X-int: -2.2549
Y-int: 1.91444 +/- 0.25885
m: 0.84901 +/- 0.12393
Dm: 0.00556
r: 0.95064

Data for Drug: C [uM]

Dose	Effect
0.375	0.024
0.75	0.053
1.5	0.128
3.0	0.184
6.0	0.656
12.0	0.92
24.0	0.939

7 data points entered.

X-int: 0.63164
Y-int: -1.0588 +/- 0.11503
m: 1.67626 +/- 0.14975
Dm: 4.28195
r: 0.98063

Data for Drug: X [uM]

Dose	Effect
0.7523	0.1417
1.505	0.229
3.01	0.623
6.02	0.797
12.04	0.9212

5 data points entered.

X-int: 0.40730
Y-int: -0.6523 +/- 0.07857
m: 1.60149 +/- 0.12267
Dm: 2.55449
r: 0.99131

Data for Non-Constant Combo: 216+T (216+T)

Dose 216	Dose T	Effect
10.0	0.0025	0.433
10.0	0.005	0.575
10.0	0.01	0.727
10.0	0.02	0.8342
10.0	0.04	0.854

5 data points entered.

Data for Non-Constant Combo: 216+C (216+C)

Dose 216	Dose C	Effect
10.0	0.75	0.123
10.0	1.5	0.244
10.0	3.0	0.4129
10.0	6.0	0.697
10.0	12.0	0.926
10.0	24.0	0.959

6 data points entered.

Data for Non-Constant Combo: 216+X (216+X)

Dose 216	Dose X	Effect
10.0	0.37625	0.132
10.0	0.7525	0.351
10.0	1.505	0.5
10.0	3.01	0.646
10.0	6.02	0.829
10.0	12.04	0.935

6 data points entered.

Dose-Effect Curve for Drugs

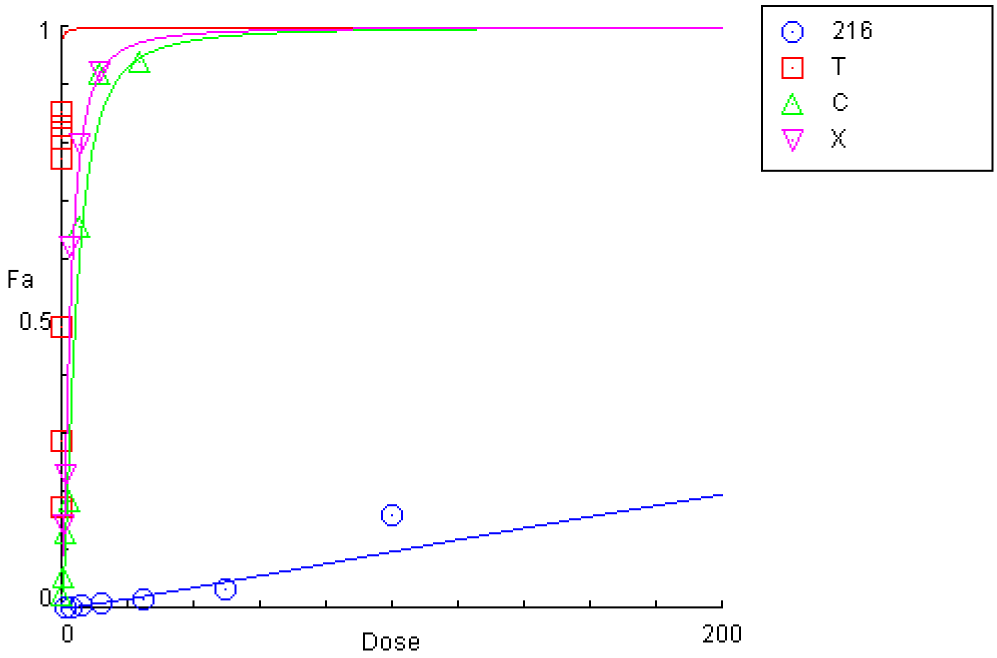

Dose-Effect Curve for Drug Combos

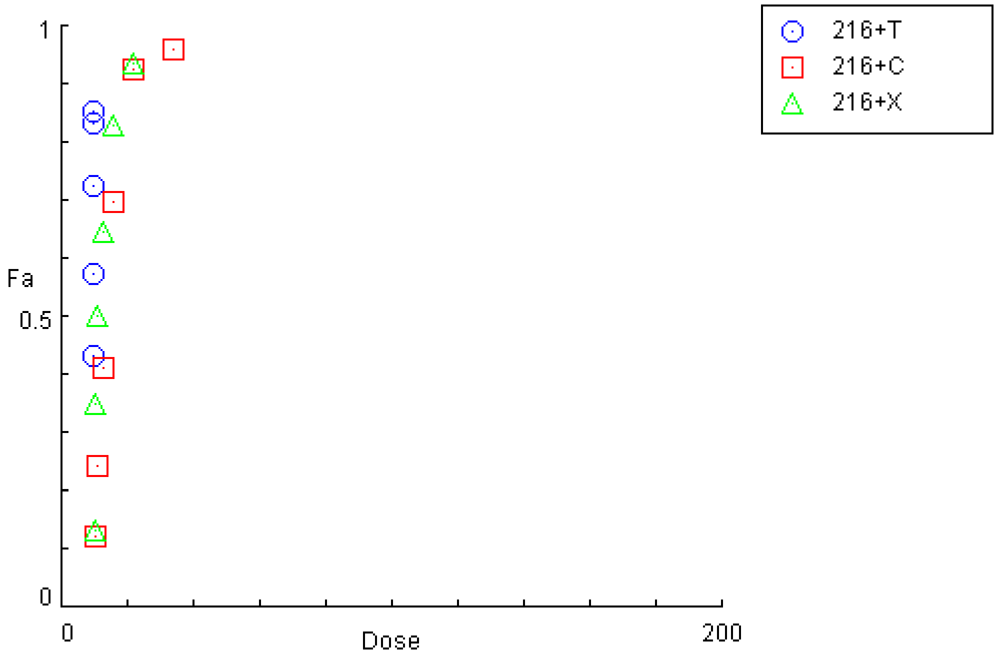

Median-Effect Plot for Drugs

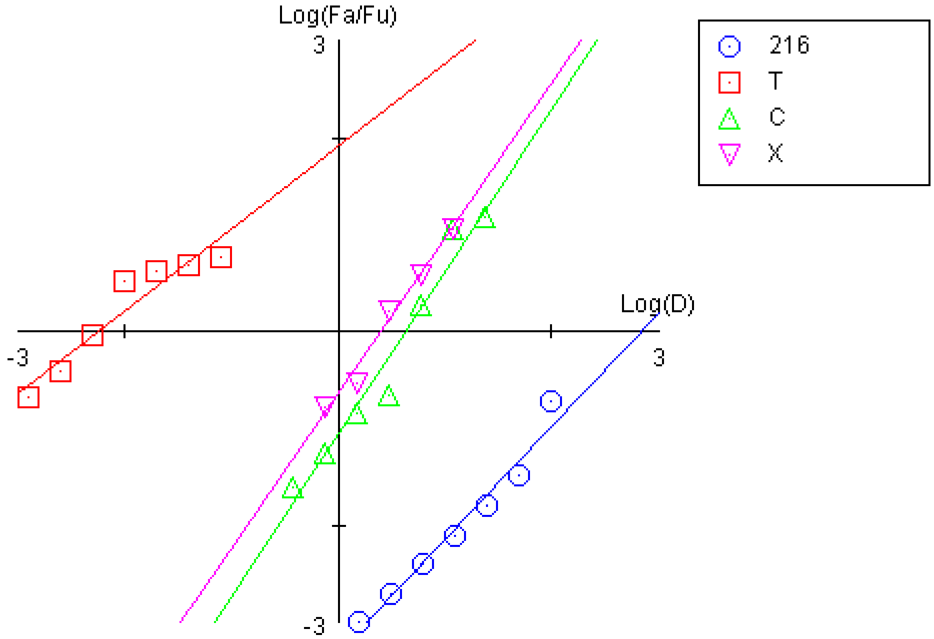

Median-Effect Plot for Drug Combos

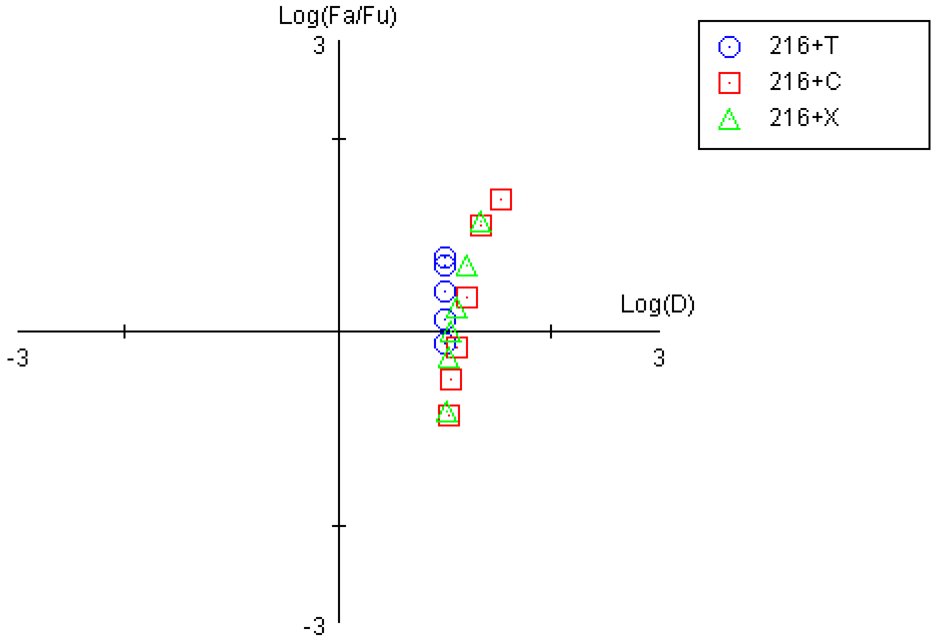

CI Data for Non-Constant Combo: 216+T (216+T)

Dose 216	Dose T	Effect	CI
10.0	0.0025	0.433	0.63653
10.0	0.005	0.575	0.64145
10.0	0.01	0.727	0.57389
10.0	0.02	0.8342	0.54015
10.0	0.04	0.854	0.90167

CI Data for Non-Constant Combo: 216+C (216+C)

Dose 216	Dose C	Effect	CI
10.0	0.75	0.123	0.64536
10.0	1.5	0.244	0.72704
10.0	3.0	0.4129	0.88452
10.0	6.0	0.697	0.85982
10.0	12.0	0.926	0.62243
10.0	24.0	0.959	0.85579

CI Data for Non-Constant Combo: 216+X (216+X)

Dose 216	Dose X	Effect	CI
10.0	0.37625	0.132	0.55207
10.0	0.7525	0.351	0.45768
10.0	1.505	0.5	0.60412
10.0	3.01	0.646	0.81832
10.0	6.02	0.829	0.88335
10.0	12.04	0.935	0.89343

Combination Index Plot

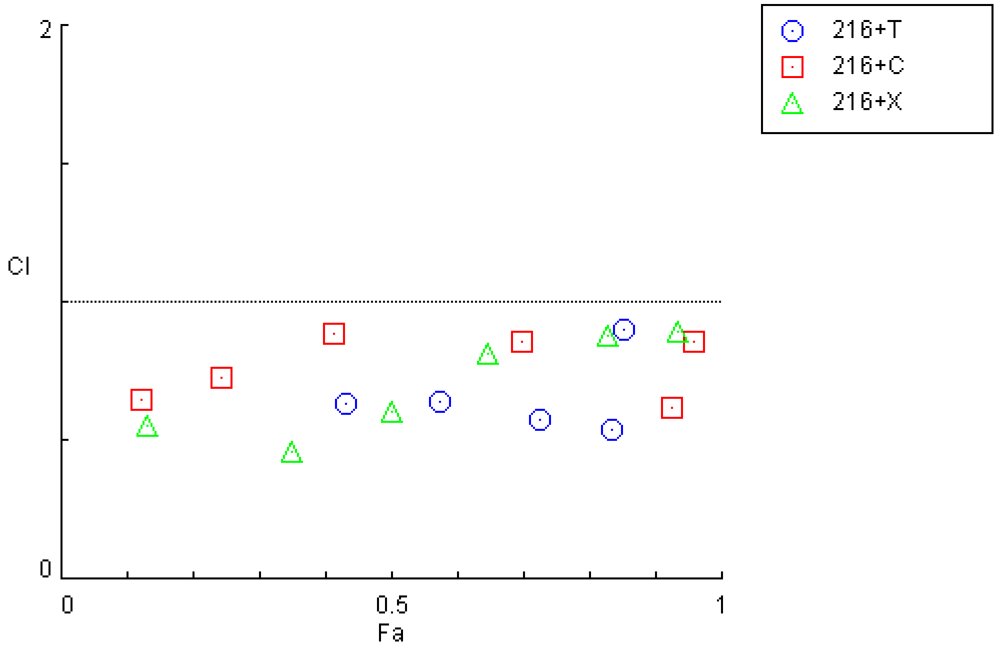

DRI Data for Non-Constant Combo: 216+T (216+T)

Fa	Dose 216	Dose T	DRI 216	DRI T
0.433	531.033	0.00405	53.1033	1.61890
0.575	865.098	0.00794	86.5098	1.58759
0.727	1541.78	0.01762	154.178	1.76240

Fa	Dose 216	Dose T	DRI 216	DRI T
0.8342	2653.43	0.03729	265.343	1.86436
0.854	3017.39	0.04453	301.739	1.11315

DRI Data for Non-Constant Combo: 216+C (216+C)

Fa	Dose 216	Dose C	DRI 216	DRI C
0.123	125.048	1.32652	12.5048	1.76869
0.244	254.652	2.18096	25.4652	1.45398
0.4129	494.990	3.47093	49.4990	1.15698
0.697	1360.71	7.03839	136.071	1.17307
0.926	5773.80	19.3331	577.380	1.61109
0.959	9846.28	28.0777	984.628	1.16991

DRI Data for Non-Constant Combo: 216+X (216+X)

Fa	Dose 216	Dose X	DRI 216	DRI X
0.132	133.989	0.78807	13.3989	2.09454
0.351	395.601	1.74030	39.5601	2.31269
0.5	668.408	2.55449	66.8408	1.69733
0.646	1116.75	3.71895	111.675	1.23553
0.829	2570.66	6.84507	257.066	1.13706
0.935	6502.86	13.4994	650.286	1.12121

DRI Plot for Non-Constant Combo: 216+T (216+T)

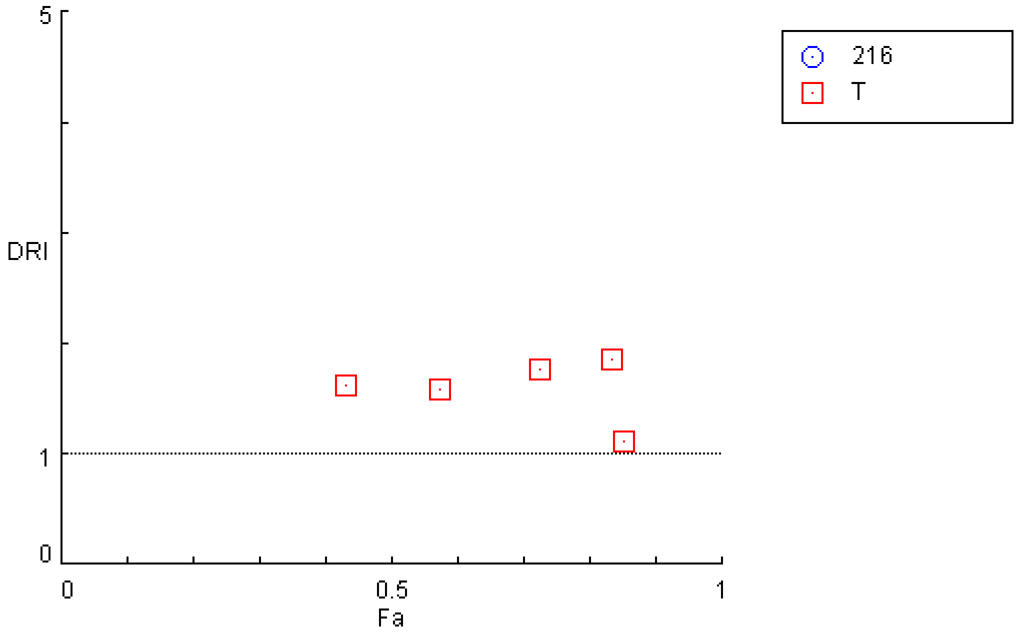

DRI Plot for Non-Constant Combo: 216+C (216+C)

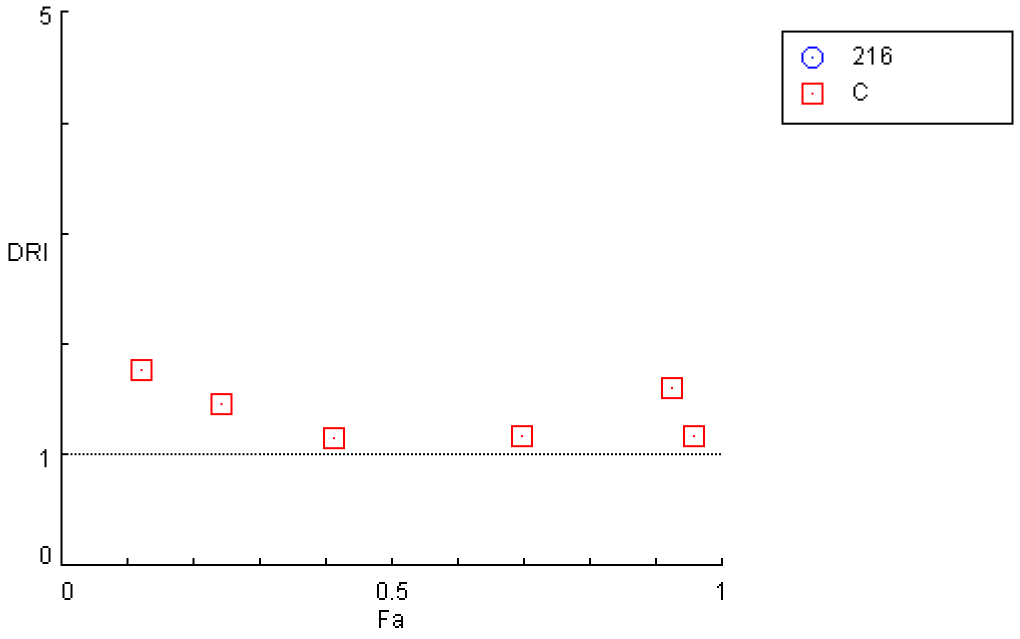

DRI Plot for Non-Constant Combo: 216+X (216+X)

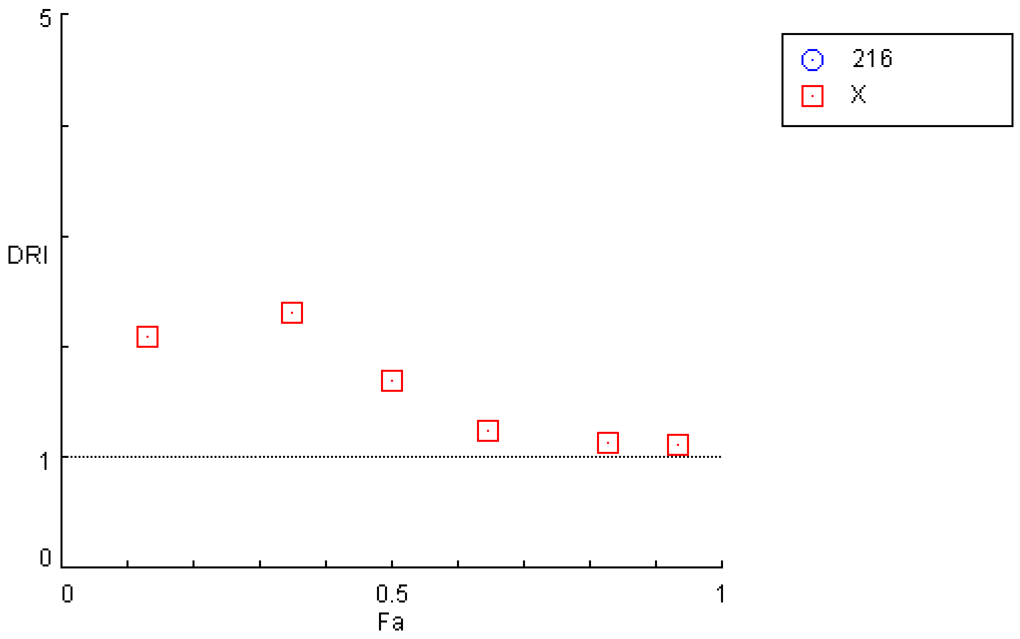

Normalized Isobologram for Combo: 216+T (216+T)

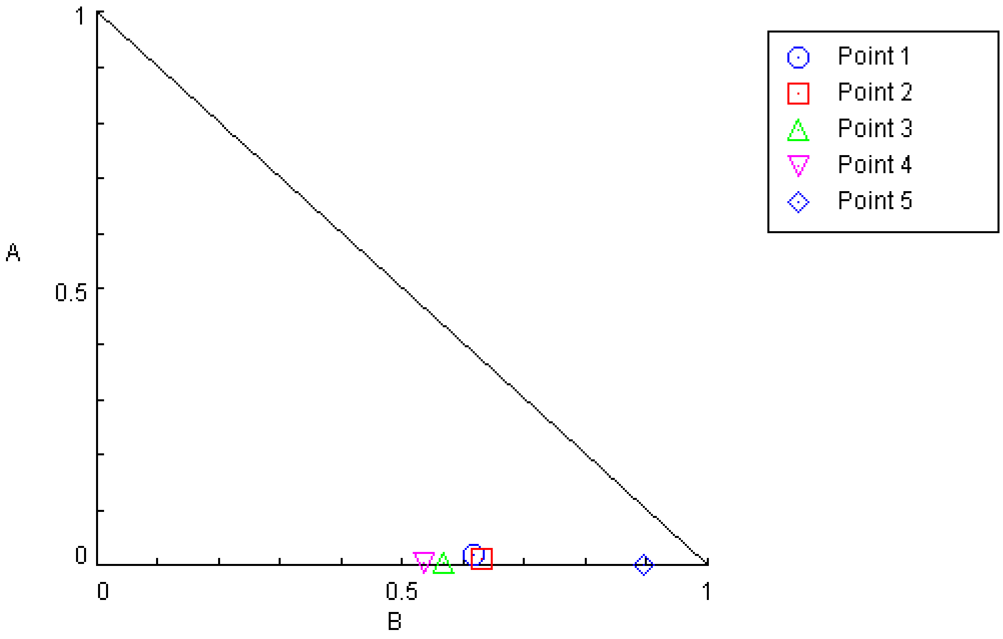

Normalized Isobologram for Combo: 216+C (216+C)

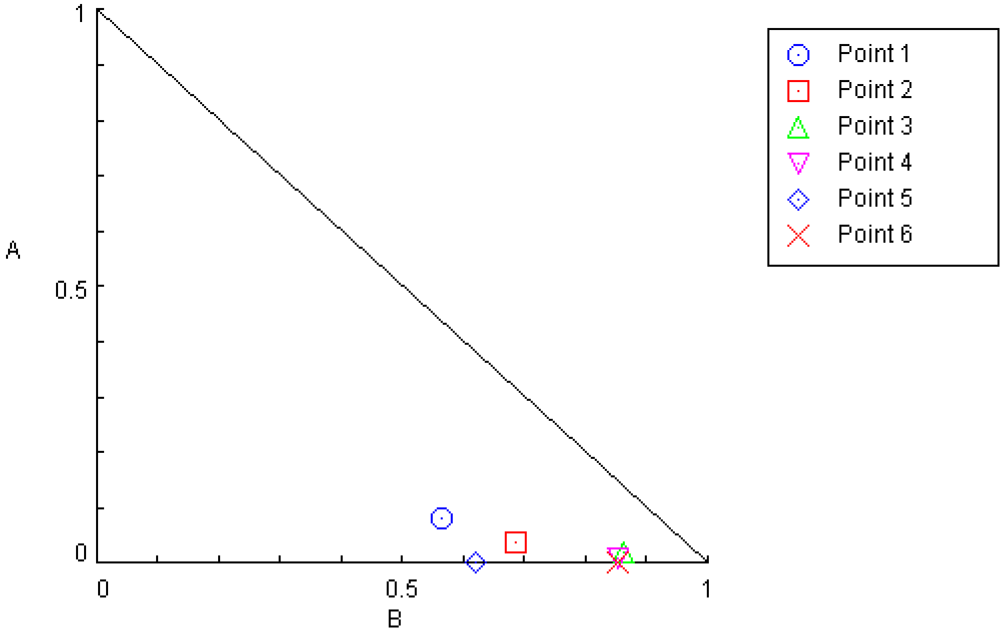

Normalized Isobologram for Combo: 216+X (216+X)

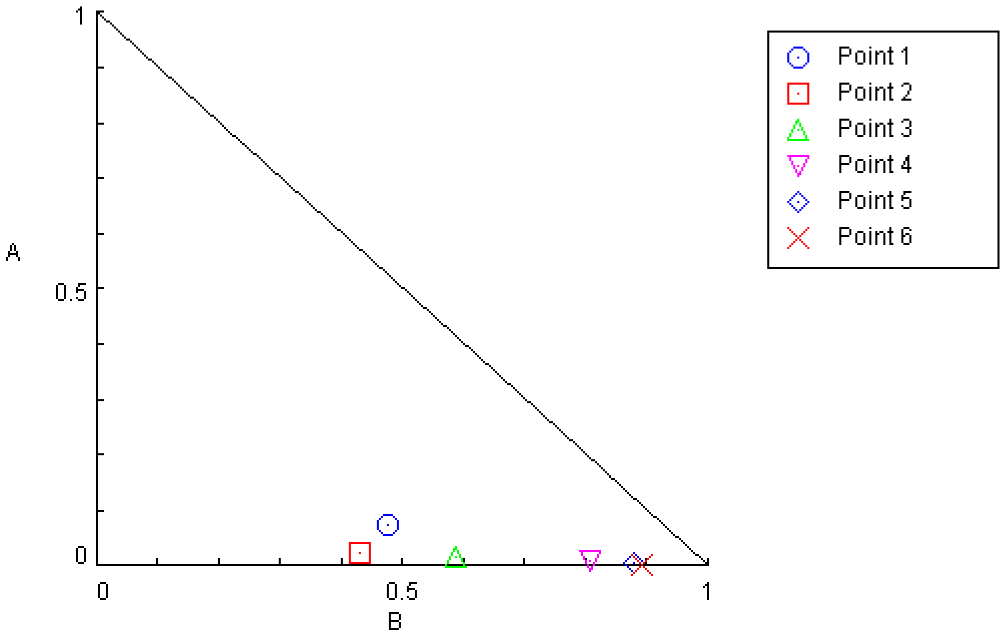

Summary Table

**Experiment Name:**	low 216 triple
**Date:**	101113
**File Name:**	C:\Users\ychen7\Dropbox\Yi\Yi\data\sulfarodamine assay\216\216 with T and C\Low 216 triple drugs 101113.cse
**Description**	

**Drug:**	216 (216) [ug/ml]
**Drug:**	taxol (T) [uM]
**Drug:**	cisplatin (C) [uM]
**Drug:**	T+C (X) [uM]
**Drug Combo:**	216+T (216+T) (216+T)
**Drug Combo:**	216+C (216+C) (216+C)
**Drug Combo:**	216+X (216+X) (216+X)

Drug/Combo	Dm	m	r
216	668.408	1.17189	0.98409
T	0.00556	0.84901	0.95064
C	4.28195	1.67626	0.98063
X	2.55449	1.60149	0.99131

CI values at:

Combo	ED50	ED75	ED90	ED95

Data for Fa = 0.5

Drug/Combo	CI value	Dose 216	Dose T	Dose C	Dose X
216		668.408			
T			0.00556		
C				4.28195	
X					2.55449

Data for Fa = 0.75

Drug/Combo	CI value	Dose 216	Dose T	Dose C	Dose X
216		1706.79			
T			0.02028		
C				8.24662	
X					5.07255

Data for Fa = 0.9

Drug/Combo	CI value	Dose 216	Dose T	Dose C	Dose X
216		4358.33			
T			0.07397		
C				15.8822	
X					10.0728

Data for Fa = 0.95

Drug/Combo	CI value	Dose 216	Dose T	Dose C	Dose X
216		8245.81			
T			0.17834		
C				24.8029	
X					16.0612

Data for Fa = 0.97

Drug/Combo	CI value	Dose 216	Dose T	Dose C	Dose X
216		12979.6			
T			0.33359		
C				34.0603	
X					22.3849
