# Supplementary material for: Ovarian carcinoma glyco-antigen targeted by human IgM antibody
Source: PLoS One. 2017 Dec 21;12(12):e0187222. doi: 10.1371/journal.pone.0187222 (PMC5739388; doi:10.1371/journal.pone.0187222)
Supplement: S2 Dataset — (ZIP) [file pone.0187222.s007.zip › FACS K & S/pt K & S.rtf]

Name	Statistic	#Cells	AnnotationTube_002 		73843	pt K conTube_003 		79245	pt k 216Tube_004 		38718	pt k 4˚conTube_005 		51057	pt k 4˚216Tube_001 		100000	pt S conTube_002 		100000	pt S 216
