# Supplementary material for: Ovarian carcinoma glyco-antigen targeted by human IgM antibody
Source: PLoS One. 2017 Dec 21;12(12):e0187222. doi: 10.1371/journal.pone.0187222 (PMC5739388; doi:10.1371/journal.pone.0187222)
Supplement: S2 Dataset — (ZIP) [file pone.0187222.s007.zip › FACS O3 /O3 staining.rtf]

Name	Statistic	#Cells	AnnotationOVCAR3 _sph+216004.fcs		35250	O3 stainOVCAR3_Ep 216l008.fcs		43553	OVCAR3_Ep control007.fcs		57407	
