# Supplementary material for: Ovarian carcinoma glyco-antigen targeted by human IgM antibody
Source: PLoS One. 2017 Dec 21;12(12):e0187222. doi: 10.1371/journal.pone.0187222 (PMC5739388; doi:10.1371/journal.pone.0187222)
Supplement: S2 Dataset — (ZIP) [file pone.0187222.s007.zip › FACS pt B2/pt. B2.rtf]

Name	Statistic	#Cells	AnnotationTube_001 control 		99900	coldTube_002 216 		82971	coldTube_001 control 		95634	37Tube_004 216 only 		94149	37Tube_001 		153846	stainingTube_002 		101277	staining
