# Supplementary material for: Ovarian carcinoma glyco-antigen targeted by human IgM antibody
Source: PLoS One. 2017 Dec 21;12(12):e0187222. doi: 10.1371/journal.pone.0187222 (PMC5739388; doi:10.1371/journal.pone.0187222)
Supplement: S2 Dataset — (ZIP) [file pone.0187222.s007.zip › O3 endo beta/O3 & endo.rtf]

Name	Statistic	#Cells	Annotation27. ovcar3 spheroid		12165	no st37. ovcar3 spheroid+endo-B		12523	no st21. ovcar3 spheroids		20850	21621. ovcar3 spheroids		30270	21631. ovcar3 spheroids+endo-B		30135	21622. ovcar3 spheroids		25306	control32. ovcar3 spheroids+endo-B		25651	control
