# Supplementary material for: Ovarian carcinoma glyco-antigen targeted by human IgM antibody
Source: PLoS One. 2017 Dec 21;12(12):e0187222. doi: 10.1371/journal.pone.0187222 (PMC5739388; doi:10.1371/journal.pone.0187222)
Supplement: S3 Dataset — (ZIP) [file pone.0187222.s008.zip › FACS pts G&M/FACS pts G & M.rtf]

Name	Statistic	#Cells	Annotation197 beads 		5000	1 		100000	Pt G PI only2 		100000	Pt g con 37˚ 		95480	Pt G 216 37˚ 		121024	Pt G con 4˚ 		88753	Pt G 216 4˚ 		156705	Pt G stain1 My 		181474	Pt M Pi only2 My 		165013	Pt M con 37˚ My 		208785	Pt M 216 37˚ My 		199919	Pt M con 4˚ My 		187643	Pt M 216 4˚
