# Supplementary material for: Ovarian carcinoma glyco-antigen targeted by human IgM antibody
Source: PLoS One. 2017 Dec 21;12(12):e0187222. doi: 10.1371/journal.pone.0187222 (PMC5739388; doi:10.1371/journal.pone.0187222)
Supplement: S4 Dataset — (ZIP) [file pone.0187222.s009.zip › FACS pt b/pt b.rtf]

Name	Statistic	#Cells	Annotationfile=2-1.fcs		300000	control pt Bfile=4-1.fcs		63325		stainfile=2-2.fcs		307075	216 pt. bfile=2-3 (1).fcs	316700	IgM controlfile=2-5.fcs		325175	control pt B
