# Supplementary material for: Ovarian carcinoma glyco-antigen targeted by human IgM antibody
Source: PLoS One. 2017 Dec 21;12(12):e0187222. doi: 10.1371/journal.pone.0187222 (PMC5739388; doi:10.1371/journal.pone.0187222)
Supplement: S5 Dataset — (ZIP) [file pone.0187222.s010.zip › FACS pt S, M, F/Pt S, M, F.rtf]

Name	Statistic	#Cells	AnnotationTube_001 216 cold 		100000	Pt STube_002 control cold 		149695	Pt STube_002 staining 		100000	Pt M stainTube_003 staining 		100000	Pt M stainTube_004 staining 		100000	Pt M stainTube_001 control 37 		268590	Pt FTube_003 216 37 		251510	Pt FTube_004 control cold 		302260	Pt FTube_005 216 cold 		189002	Pt F
