# Supplementary material for: Ovarian carcinoma glyco-antigen targeted by human IgM antibody
Source: PLoS One. 2017 Dec 21;12(12):e0187222. doi: 10.1371/journal.pone.0187222 (PMC5739388; doi:10.1371/journal.pone.0187222)
Supplement: S6 Dataset — (ZIP) [file pone.0187222.s011.zip › FACS Pt C/Pt C.rtf]

Name	Statistic	#Cells	AnnotationUntitled 1. unstained C Ascites		18345	Pt c no stUntitled 2. C Ascites		47152	Pt C stainedUntitled 7. C Ascites mAb 216		100000	Pt C 216Untitled 8. C Ascites mAb MS2B6		100000	Pt C control
