# Supplementary material for: Ovarian carcinoma glyco-antigen targeted by human IgM antibody
Source: PLoS One. 2017 Dec 21;12(12):e0187222. doi: 10.1371/journal.pone.0187222 (PMC5739388; doi:10.1371/journal.pone.0187222)
Supplement: S6 Dataset — (ZIP) [file pone.0187222.s011.zip › FACS Pt W /pt W.rtf]

Name	Statistic	#Cells	Annotation197 beads 		5000	pt WTube_001 control 		146041	37˚ube_004 216 only 		138632	37˚ube_001 control 		139314	coldTube_002 216 only 		136493	coldTube_001 		85870	stainTube_002 		200000	stainTube_003 		135160	
